# Morbidity, mortality, and risk factors of emergency colorectal surgery among older patients in the Acute Care Surgery service: A retrospective study

**DOI:** 10.1016/j.amsu.2020.11.001

**Published:** 2020-11-06

**Authors:** Chonlada Krutsri, Preeda Sumpritpradit, Pongsasit Singhatas, Tharin Thampongsa, Samart Phuwapraisirisan, Goragoch Gesprasert, Jakrapan Jirasiritham, Pattawia Choikrua

**Affiliations:** a Division of Trauma Surgery and Surgical Critical Care, Department of Surgery, Faculty of Medicine, Ramathibodi Hospital, Mahidol University, Bangkok, Thailand; b Division of General Surgery, Department of Surgery, Faculty of Medicine, Ramathibodi Hospital, Mahidol University, Bangkok, Thailand; c Division of Vascular and Transplant, Department of Surgery, Faculty of Medicine, Ramathibodi Hospital, Mahidol University, Bangkok, Thailand; d Surgical Research Unit, Faculty of Medicine, Ramathibodi Hospital, Mahidol University, Bangkok, Thailand

**Keywords:** Colorectal emergency, Colorectal surgery, Colorectal perforation, Emergency surgery, Acute care surgery

## Abstract

**Background:**

Acute Care Surgery (ACS) is a rapid response system in emergency surgical conditions. The patients who over 60 year-old have numerous factors associated with high mortality and morbidity in emergency colorectal surgery. We aimed to identify potentially preventable risk factors, to improve patients’ outcomes.

**Methods:**

A retrospective review of patients age over 60 year-old undergoing emergency colorectal surgery in the ACS service from August 1, 2017 through November 30, 2019.

**Results:**

Ninety-two patients were analyzed, average age 72.41 years. The most common diagnosis was complicated colorectal cancer (76, 83.52%) with locations on the right (37, 41.51%), left (35,39.33%), and rectum (17, 19.10%). Clinical presentations were obstruction without perforation (61, 67.03%), perforation (25, 27.17%), and ischemia (2, 2.17%). Overall mortality was 6.52%. Cause of death included septic shock (3, 50%); respiratory failure (3, 50%); and pulmonary embolism (1, 16.67%). Morbidity from surgical and medical complications were 41.30% and 26.08%, respectively. For all causes, operations included resection with primary anastomosis (62, 71.26%); Hartmann's operation (11, 12.64%); and loop colostomy (12, 13.79%). Average operative time was 159.86 min. In emergency colorectal surgery, pre-existing heart disease, clinical perforation, and ventilator dependency increased risk of death 7.6-, 16.5-, and 0.08-fold, respectively.

**Conclusion:**

Clinical perforation leads to sepsis and septic shock in older patients, this may be modifiable to improve mortality by developing an early, rapid, protocol-driven surgical sepsis fast-track process. Ventilator dependency is potentially modifiable with postoperative advanced surgical critical care. The non-modifiable risk factor of co-morbid heart disease might be improved by postoperative advanced critical care for close monitoring.

## Highlights

• Most of emergency colorectal surgeries resulted from complications of colorectal cancer; age was not a contraindication for resection with primary anastomosis.
• Clinical perforation leads to sepsis and septic shock in older patients. This may be modifiable to improve mortality by developing an early, rapid, protocol-driven surgical sepsis fast-track process.
• Ventilator dependency is potentially modifiable with postoperative advanced surgical critical care.
• Preoperative nutrition status, other comorbidities, preoperative anticoagulant use, location of disease, operative time, intraoperative fluid replacement, and blood loss were not risk factors of increased mortality.

## 1 Background

The Acute Care Surgery service system is a rapid surgical response system for definite surgery or damage-control surgery under emergency surgical conditions that obviously improves patient outcome [1,2]. Older patients who age over 60 year-old undergo colorectal surgery have numerous factors that are associated with high rates of mortality and morbidity, especially in emergency settings [3]. Emergency colorectal surgery is performed in one-fourth of all colorectal surgeries, with a 10%–25% mortality rate and 30%–50% morbidity rate in all age groups [4,5]. The challenges to care in people who age over 60 year-old are great because of higher risks involved in surgery among patients with pre-existing co-morbidities and low functional reserves, leading to higher rates of postoperative complications and mortality than in younger patients [6]. Therefore, considering the high incidence of morbidity and death after emergency colorectal surgery, it is important to determine risk factors involved in emergency colorectal surgical care, to improve patients outcomes. In this study, we aimed to identify potentially preventable or modifiable risk factors that impact mortality and morbidity if handled properly, which might improve outcomes in older patients who over 60 year-old undergoing emergency colorectal surgery.

## 2 Methods

We collected retrospective data of patients age >60 years who had emergency colorectal surgery in the Acute Care Surgery service of a tertiary hospital, from August 1, 2017 through November 30, 2019. We use the definition of older patients is > 60 years-old in developing countries, according to the World Health Organization definition. Demographic data, co-morbidities, hospital course, time to the operating room (OR) for source control surgery, operative details, intraoperative fluid replacement, blood loss, operative time, and postoperative surgical and medical complications were collected from medical records.

The time to operation room (OR) is defined as time from diagnosis until the time to start the operation, which is guaranteed to be within 180 min in quality control of the Acute Care Surgery service at the hospital. Every case in the Acute Care Surgery service is first attended by a second-year general surgery resident with the guaranteed time arrival within 30 min, regarding to quality control of Acute Care Surgery service, after consultation by an emergency physician. In case of septic shock or cardiac arrest immediately consultation to chief (fourth-year) general surgery resident is mandate and care should be taken within 10 min according to our service's quality control. Every surgery performed or covered is carried out by general surgery and trauma staffs in Acute Care Surgery team, who had 3–8 years working experience after graduated of general surgical training and are not graduate of colorectal fellowship training. Acute Care Surgery service of our hospital, tertiary hospital, has a standard quality control of time; consultation time is within 30 min and time to operation room is within 180 min.

Preoperative venous thromboembolism (VTE) is assessed in every case, use Caprini deep vein thrombosis (DVT) risk score; but administration of antiplatelets prophylaxis such as unfractionated or low-molecular-weight heparin when risk score is reach were according to surgeon's decision in emergency setting owing to a risk of intra- and postoperative bleeding. Postoperative Enhanced Recovery after Surgery (ERAS) protocols are applied for all patients within 3 days after surgery.

Statistical analysis was conducted using Stata 14.2 software (StataCorp LLC, College Station, TX, USA). Continuous variables are summarized using mean, standard deviation, and median. Categorical variables are summarized using chi-square and presented as percentage. All descriptive statistics were stratified by mortality and morbidity. Logistic regression was used to estimate the odds ratio and 95% confidence interval (CI) for risk factors.

## 3 Results

A total 92 patients who over 60 year-old and had emergency colorectal surgery were included and analyzed in our study. Table 1 shows a summary of demographic data. The average patient age was 72.41 years, with 46 (50%) men and 46 (50%) women. Average length of stay (LOS) in the hospital was 8 days or longer if postoperative intensive care (ICU) was required. The most common co-morbidities were hypertension (HT), 60 patients (65.22%); diabetes mellitus (DM), 30 (32.61%); dyslipidemia (DLP), 25 (27.17%); ischemic or hemorrhagic stroke, 17 (18.48%); and congestive heart disease, previous myocardial ischemia, or arrythmia, 13 (14.13%). Prior to surgery, 18 (19.57%) patients were taking anticoagulants or anti-platelets which prone to have intra- and post-operative bleeding. The average Caprini DVT risk score in pre-operative assessment was 4, which is compatible with moderate risk for VTE. An intermittent pneumatic compression (IPC) device was used intra- and postoperatively in all patients who had no history of previous deep vein thrombosis. In a subgroup analysis between patients who survived and those who died, we found that Caprini risk score was significantly related to death, with a score of 5 in patients who died and 4 in those who survived (p = 0.042). Post-operative pulmonary embolism was the cause of death in 1 patient (16.67%) which pre-operative Caprini risk score of 5 and did not receive pre-operative anti-platelets due to risk of intra- and postoperative bleeding in septic shock. The median time to an OR was 60 min, which is within the guarantee of our quality control. Twenty (21.74%) patients had septic shock on admission, which was the cause of death in 50% of emergency colorectal surgeries in this study. Pre-operative nutrition status assessment by nutrition alert form (NAF) was normal in 35 patients (49.30%), 25 (35.21%) had moderate malnutrition, and 11 (15.49%) had severe malnutrition; preoperative nutrition status was not a risk factor of increased mortality: odds ratio 1.858 (95% CI 0.50–6.86), p = 0.352 (Table 3).

**Table 1:** Characteristics of patients undergoing emergency colorectal surgery, age over 60 year-old.

		Died N = 6	Survived N = 86	Total N = 92	p-value
Sex	Male	1 (16.67)	45 (52.33)	46 (50)	0.203
	Female	5 (83.33)	41 (47.67)	46 (50)	
Age (y), mean (standard deviation)		83.5 (10.46)	71.63 (10.42)	72.41 (10.78)	***0.008***
Total length of stay (days), median (P25, P75)		13.5 (6, 25)	8 (5, 17)	8 (6, 17)	0.295
Time to operating room (mins)		80 (60,120)	60 (60,120)	60 (60,120)	0.907
Comorbidities, n (%)	Arterial hypertension	4 (66.67)	56 (65.12)	60 (65.22)	0.999
	Diabetes mellitus	2 (33.33)	28 (32.56)	30 (32.61)	0.999
	Dyslipidemia	3 (50)	22 (25.58)	25 (27.17)	0.339
	Stroke	2 (33.33)	15 (17.44)	17 (18.48)	0.306
	Heart disease	3 (50)	10 (11.63)	13 (14.13)	***0.035***
	Renal insufficiency	2 (33.33)	13 (15.12)	15 (16.30)	0.252
	Other disease*	2 (33.33)	15 (17.44)	17 (18.48)	
Anticoagulants/antiplatelets, n (%)		2 (33.33)	16 (18.60)	18 (19.57)	0.334
Caprini deep vein thrombosis risk score, median (P25, P75)		5 (5, 6)	4 (4, 5)	4 (4, 5)	***0.042***
Sepsis/septic shock, n (%)		3 (50)	17 (19.77)	20 (21.74)	0.114
Preoperative nutrition status, n (%)	Normal	2 (50)	33 (49.25)	35 (49.3)	0.100
	Moderate malnutrition	0	25 (37.31)	25 (35.21)	
	Severe malnutrition	2 (50)	9 (13.43)	11 (15.49)	
Overall mortality (%)				6.52%	
Cause of death, n (%)	Septic shock	3 (50)			
	Respiratory failure	3 (50)			
	Cardiogenic cause	1 (16.67)			
	Pulmonary embolism	1 (16.67)			
	Disease process	1 (16.67)			

* are asthma, chronic obstruction pulmonary disease, chronic kidney disease, autoimmune disease, and liver cirrhosis.

The overall mortality rate in patients who had emergency colorectal surgery with age over 60 year-old was 6.52%. The cause of death were septic shock in 3 patients (50%), respiratory failure in 3 (50%), cardiogenic cause in 1 (16.67%), pulmonary embolism in 1 (16.67%), and disease process in 1 (16.67%) patient, which overlap cause in 3 patients. Morbidity from preexisting heart disease led to postoperative myocardial infarction in 1 (1.09%) patient, congestive heart failure in 6 (6.52%), and arrhythmia in 2 (2.17%) patients; these data are not shown in the table.

Table 2 presents clinical data and operative details. The most common reasons for emergency colorectal surgery were complicated colorectal cancer in 76 (83.52%) patients, complicated diverticulitis in 11 (12.09%), and sigmoid volvulus in 2 (2.2%) patients. The most common location of disease was the right side in 37 (41.57%), left side in 35 (39.33%), and rectum in 17 (20.24%) patients. Initial presentation was obstruction without perforation in 61 (67.03%), perforation in 25 (27.17%), ischemia in 2 (2.17%), and bleeding in 3 (3.26%) patients. In subgroup analysis, we found that initial presentation of perforation was significantly associated with a high rate of death, 83.33% in patients who died versus 23.26% in those who survived (p = 0.005). The procedures performed included resection with primary anastomosis in 62 (71.26%) patients with both right- and left-side lesions, Hartmann's operation in 11 (12.64%), loop colostomy in 12 (13.79%), and percutaneous drainage in 2 (2.3%) patients. The average preoperative lactate level was 2.35 mmol/L, which was not reach statistically significant in patients who died and in those who survived: 15.9 and 2.3 mmol/L, respectively (p = 0.117). The average operative time and blood loss was 159.86 min and 150 mL, respectively. The median intra-operative fluid replacement was 1550 mL, which was not significantly different between patients who died and those who survived (p = 0.141). Postoperative intensive care (ICU) was required in 26 (28.89%) patients, with average LOS in the ICU of 4.5 days. LOS in the ICU was significantly higher in patients who died, 9.5 days versus 4.08 days in survivors (p = 0.020). The most common indication for an ICU requirement was because of high American Society of Anesthesiologists (ASA) physical status classification 3–4 in 13 (14.13%) patients, preoperative septic shock in 12 (13.04%), respiratory failure in 8 (8.70%), and co-morbid heart disease in 4 (4.35%) patients. A total 29 (32.22%) patients in the ICU were ventilator-dependent, which was significantly higher in patients who died (83.33%) than in those who survived (28.57%), p = 0.012. The rate of surgical complications was 41.30%, which comprised surgical site infection (SSI) in 15 (16.30%) patients, postoperative ileus in 8 (8.70%), anastomosis leakage in 4 (4.35%), and intra-abdominal collection in 4 (4.35%).

**Table 2:** Surgical disease and operative details of emergency colorectal surgery in patients age over 60 year-old.

		Death N = 6	Alive N = 82	Total N = 92	p-value
Cause of disease, n (%)	Colorectal cancer	4 (66.67)	72 (84.71)	76 (83.52)	0.367
	Complicated diverticulitis	2 (33.33)	9 (10.59)	11 (12.09)	
	Sigmoid volvulus	0	2 (2.35)	2 (2.2)	
	Rectal ischemia	0	1 (1.18)	1 (1.1)	
	Metastasis	0	1 (1.18)	1 (1.1)	
Location of disease	Right side	1 (20)	36 (42.86)	37 (41.57)	0.207
n (%)	Left side	4 (80)	31 (36.9)	35 (39.33)	
	Rectum	0	17 (20.24)	17 (20.24)	
Initial presentation	Obstruction	0	61 (71.76)	61 (67.03)	0.001
n (%)	Perforation	5 (83.33)	20 (23.26)	25 (27.17)	***0.005***
	Ischemia	1 (16.67)	1 (1.16)	2 (2.17)	0.127
	Bleeding	0	3 (3.49)	3 (3.26)	0.999
Procedure	Resection with primary anastomosis	4 (100)	58 (69.88)	62 (71.26)	0.999
n (%)	Hartmann's operation	0	11 (13.25)	11 (12.64)	
	Loop colostomy	0	12 (14.46)	12 (13.79)	
	Percutaneous drainage and antibiotics	0	2 (2.41)	2 (2.3)	
Postoperative intensive care (ICU) requirement, n (%)		2 (33.33)	24 (28.57)	26 (28.89)	0.999
Length of stay in ICU (days), mean (standard deviation, SD)		9.5 (6.36)	4.08 (2.73)	4.5 (3.26)	***0.020***
Indication for ICU	Older age	0	13 (15.12)	13 (14.13)	0.589
n (%)	Septic shock	2 (33.33)	10 (11.63)	12 (13.04)	0.174
	Respiratory failure	1 (16.67)	7 (8.14)	8 (8.70)	0.430
	Comorbid heart disease	0	4 (4.65)	4 (4.35)	0.999
Postoperative ventilator dependency, n (%)		5 (83.33)	24 (28.57)	29 (32.22)	***0.012***
Operative time (min), mean (SD)		134.16 (68.51)	161.76 (71.81)	159.86 (71.55)	0.365
Intraoperative fluid replacement (mL), median (P25, P75)		925 (550, 1700)	1600 (1000, 2400)	1550 (1000, 2400)	0.141
Intraoperative blood loss (mL), median (P25, P75)		75 (50, 300)	150 (50, 300)	150 (50, 300)	0.429
Preoperative lactate level (mmol/mL)		15.9	2.3	2.35	0.117
Morbidity from surgical complication (%)				41.30%	
Surgical complications, n (%)	Surgical site infection	1 (16.67)	14 (16.28)	15 (16.30)	0.999
	Postoperative ileus	1 (16.67)	7 (8.14)	8 (8.70)	0.430
	Anastomosis leakage	1 (16.67)	3 (3.49)	4 (4.35)	0.240
	Collection	0	4 (4.65)	4 (4.35)	0.999

Univariate analysis of risk factors is shown in Table 3, according to odds ratios. Identified risk factors of increase mortality and morbidity in emergency colorectal surgery were co-morbid heart disease, clinical perforation, and ventilator dependency, with a 7.6-, 16.5-, and 0.08-fold increased risk of death, respectively (p < 0.05). The time from diagnosis to the OR, operative time, intra-operative intravenous fluid replacement, blood loss, preoperative nutritional status, preoperative lactate level, postoperative complications, and other co-morbidities were not risk factors of death.

**Table 3:** Univariable analysis of risk factors for morbidity and mortality of patients age over 60 year-old who's undergoing emergency colorectal surgery.

	Odds ratio (95% CI)	p-value
Comorbid heart diseases	7.6 (1.35–42.90)	***0.022***
Location of disease	1.05 (0.315–3.49)	0.939
Colorectal perforation	16.50 (0.82–149.59)	***0.013***
Time to operating room	1.00 (0.99–1.01)	0.900
Operative times	0.99 (0.98–1.01)	0.360
Intraoperative blood loss	0.99 (0.99–1.00)	0.379
Intraoperative fluid replacement	0.999 (0.99–1.00)	0.223
Preoperative nutritional status	1.858 (0.50–6.86)	0.352
Anastomosis leakage	5.53 (0.48–63.26)	0.169
Postoperative intensive care requirement	0.80 (0.14–4.66)	0.804
Postoperative ventilator dependency	0.08 (0.008–0.72)	***0.024***

## 4 Discussion

Life expectancy in emergency abdominal surgery has increased, including among patients of any age. However, regardless of age, emergency surgery is associated with a 3- to 10-fold increased rate of operative mortality; emergency colorectal surgery has a 9-fold increased risk of death [7,8]. A previous study found that age is not an absolute contraindication in standard colorectal surgery of emergency setting; however, postoperative complications, mortality, and morbidity increase with age because of low physiological reserve and pre-existing co-morbidities, which affect patient outcome [9]. Acute Care Surgery is a service system for emergency general surgery in our tertiary hospital, with guarantees of patients being attended within 30 min after consultation, time to the operation room is within 180 min, and attended of advanced surgical critical care for postoperative intensive care. Using this system, we can improve the number of non-trauma cases and provide good patient outcomes [1]. In this study, we sought to identify risk factors of mortality and morbidity in older patients who over 60 year-old undergoing emergency colorectal surgery, with the aim to identify modifiable risk factors and that can improve patients outcomes.

Regarding our results, co-morbid heart disease was associated with a 7.6-fold increased risk of death (p = 0.022), leading to worsening of cardiac pumping and are the risk of cardiac damage causes acute coronary heart disease and atrial fibrillation after sepsis which increase risk of death. A co-morbid heart disease was present in 50% of patients who died. This reflects that co-morbid heart disease is an unmodifiable risk factor, and preparation of these patients in emergency settings is sometimes limited. But, we can potentially improve outcomes in this group of patients by require timely operation and transfer to the ICU postoperatively for close monitoring such as a volume status and electrocardiography (ECG) for emergency cariogenic conditions such as atrial fibrillation, congestive heart failure, or myocardial infarction. A previous study among octogenarians showed a 16% probability of death from cardiovascular disease, but this was not statistically significant; also a neurological comorbidity had a 4-fold increased risk of death [7]. A neurological co-morbidities, such as ischemic or hemorrhagic stroke or even preoperative use of anticoagulants or anti-platelets which are risk of intra- and postoperative bleeding, were not associated with an increased risk of death in our study.

Our service encourages an ERAS protocol for postoperative early ambulation and assessment of preoperative VTE risk using Caprini DVT risk score in every patients, to prevent adverse fatal events of postoperative VTE such as pulmonary embolism. A previous report of a VTE prophylaxis benchmark for general surgery showed that VTE prophylaxis can reduce the incidence of pulmonary embolism, which can reduce mortality from 1.1% to 0.5% (p < 0.01) [12]. Our study showed significant differences in Caprini risk score between patients who died and survivors, 5 and 4, respectively (p = 0.042). A score of 4–5 indicates a moderate to high risk of postoperative VTE which needs mechanical and medical prophylaxis. We therefore use intermittent pneumatic pressure in all patients who had no history of DVT both intra- and postoperatively until the patients can walk; however, we do not administer anti-platelets such as unfractionated or low-molecular-weight heparin owing to the risk of intra- and postoperative bleeding in emergency setting. From our study, a high Caprini risk score was associated with increased risk of death (p = 0.042). We have 1 patient die from postoperative pulmonary embolism which preoperative Caprini risk score 5 and did not received anti-platelets because caution of intra- and post-operative bleeding. So, this is important to give anti-platelets according to preoperative Caprini risk score in patients who does not have preoperative coagulapathy to prevention of postoperative pulmonary embolism even in emergency setting.

The most frequent cause of death was septic shock, with a rate of 50%, mainly caused by colorectal perforation (83.33%). Our results showed the initial clinical presentation of perforation is associated with a 16.5-fold increased risk of death (p = 0.013), especially if perforation occurred before hospitalization more than 24 h. With 67.03% clinical presentation of obstruction without perforation, we can prevent further perforation by not delaying in surgery. In our study, the average time to operating room was 60 min. Within this 60 min, we found no further perforation with clinical obstruction, even in patients with complete obstruction and competent ileocecal valve with cecal size 9 cm in plain film abdomen. Calos et al. reported that septic shock was found 15.6% of emergency colorectal surgeries; in their study, this was the cause death in 5 patients but was not found to be a significant risk factor associated with death [3]. From our study, septic shock increases the risk of death but this may be modifiable with further development of this Acute Care Surgery service and fast-track surgical sepsis protocols to improve mortality and morbidity, which requires multidisciplinary team involve. The main etiology of emergency colorectal surgery in our study populations was complicated colorectal cancer (83.52%), the same as in previous studies [13]. Lesions were more commonly located on the right side of the colon (41.57%) than on the left side (39.33%). In our patients with either right-side, left-side, or rectal lesions, we performed resection with primary anastomosis to avoid colostomy formation, except in patients with severe fecal contamination from perforation and those with septic shock, in whom we performed Hartmann's operation (12.64%). The most frequently performed operation emergency was resection with primary anastomosis and anastomosis leakage occur only 4.35%; these rates were lower than in previous studies [3].(5–6) As seen in Table 3, anastomosis leakage was not associated with a significantly increased risk of death. This might be because anastomosis leakage can now be treated in a minimally invasive manner, such as with percutaneous drainage, bowel rest, and antibiotics. Santos et al. reported that septic shock is the leading cause of death; in their study, the most common operations were Hartmann's operation (85%), and the mortality rate was as high as 34% [14]. Compared with our results, even with increased risk of death in patients with septic shock, the overall mortality rate was only 6.52%. This implies that resection with primary anastomosis is not a contraindication in emergency colorectal surgery. Pirrera et al. reported an anastomosis leak rate of only 6.2%, which confirms that older age is not a contraindication for primary anastomosis [6]. Calos et al. supported this with a low leakage rate in resection with primary anastomosis, even in emergency settings and older patients [3]. This outcomes helps to decided the selection of patients for each operation; with primary anastomosis, patients with septic shock and severe fecal contamination are avoided and Hartmann's operation should be performed. Hence, resection with primary anastomosis is not a contraindication in emergency settings, with an anastomosis leak rate of only 4.5% and no significantly increased risk of death. Proper patients selection for each type of operation is important.

The morbidity rate from surgical complications was high at 41.30%, mainly owing to minor complications such as SSI (16.30%). Because the average time to the OR for emergency colorectal surgery was 60 min in our study, the time of source control in perforation of surgical sepsis was not delayed, which represents one factor that facilitated good outcomes. The main reasons for postoperative intensive care requirement was owing to septic shock and respiratory failure, leading to postoperative ventilator dependency. Morbidity from ventilator dependency was high (83.33%); in patients who died postoperatively, ventilator dependency had a 0.08-fold increased risk of death (p < 0.05). So, postoperative advanced surgical critical care is important role for preventing death and promoting early extubation. Preoperative nutrition status was not a significant risk factor of death, as in a previous report [3]. Average intra-operative fluid replacement was 1550 mL, which was also not a risk factor of death; however, this may cause postoperative volume overload in patients with low cardiac functional reserve which increase ventilator dependent.

So, from our study, the timely approach to the patients with surgical sepsis fast-track and a good critical care team for post-operative taking care of complicated and emergency colorectal surgery conditions especially in older patients is important to facilitate the good outcome. The limitation of this study is limited number of patients and surgical sepsis fast-track protocol is under develop, further information of morbidity and mortality after successful develop surgical sepsis fast-track should investigated. This study was reported in line with the STROCSS guideline [15].

## 5 Conclusion

In our study, most emergency colorectal surgeries resulted from complications of colorectal cancer; age was not a contraindication for resection with primary anastomosis. An early approach in these patients, from diagnosis to surgery, will facilitate a good patients outcome. Clinical perforation leads to sepsis and septic shock in older patients, which was the cause of death in our study; this may be modifiable to improve mortality by developing an early, rapid, protocol-driven surgical sepsis fast-track process. The non-modifiable risk factor of co-morbid heart disease might be improved by postoperative advanced critical care for close monitoring. Ventilator dependency is potentially modifiable with postoperative advanced surgical critical care. Preoperative nutrition status, other comorbidities, preoperative anticoagulant use, location of disease, operative time, intraoperative fluid replacement, and blood loss were not risk factors of increased mortality.

## Registration of Research Studies

1.Registry used : Thai Clinical Trails Registry

2.Unique Identifying number or registration ID : TCTR20200823002

3.Hyperlink to your specific registration :http://www.clinicaltrials.in.th/index.php?tp=regtrials&menu=trialsearch&smenu=fulltext&task=search&task2=view1&id=6539

## Strengths and limitations

Strengths of the study is evaluate the clinical risk factor than can modified to improve the patients outcome.

Limitations of this study is small number of participants.

## Ethics approval and consent to participate

The ethics was approved and permitted by Ethical committee of Mahidol University.

## Consent for publication

Not applicable.

## Availability of data and materials

https://docs.google.com/spreadsheets/d/1shFlz5H5KpUJfhfnXquttQFsd9NQ3vmMYJruqBQOO68/edit.

## Funding

No funding supported from any payers.

## Authors's contributions

CK has made substantial contributions to the conception, design of the work, interpretation of data, has drafted the work, and revised it.

PS has made substantial contributions to the conception, interpretation of data, and substantively revised it.

PS have made the acquisition and interpretation of data.

TT have made the acquisition and interpretation of data.

SP have made the acquisition and interpretation of data.

GG have made the acquisition and interpretation of data.

JJ have made the acquisition and interpretation of data.

PC has made an analysis, and interpretation of data. All authors read and approved the final manuscript.

## Provenance and peer review

Not commissioned, externally peer reviewed.

## Consent

Ethics approval was permitted from Mahidol University for consent of retrospective review patients data.

Patients’ names, initials, or hospital numbers should not be used.

## Guarantor

The Guarantor is Dr. Chonlada Krutsri who is the corresponding author of this manuscript.

## Declaration of competing interest

The authors declare that they have no competing interests.
